# Rs7206790 and rs11644943 in *FTO* Gene Are Associated with Risk of Obesity in Chinese School-Age Population

**DOI:** 10.1371/journal.pone.0108050

**Published:** 2014-09-24

**Authors:** Yuyang Xu, Jie Ling, Min Yang, Hao Wang, Shuai Zhang, Xuhui Zhang, Yimin Zhu

**Affiliations:** 1 Department of Epidemiology & Biostatistics, Zhejiang University School of Public Health, Hangzhou, China; 2 Hangzhou Center for Disease Control and Prevention, Hangzhou, China; 3 Department of Nutrition, Zhejiang University School of Public Health, Hangzhou, China; 4 Department of Pathology, Zhejiang University School of Medicine, Hangzhou, China; MOE Key Laboratory of Environment and Health, School of Public Health, Tongji Medical College, Huazhong University of Science and Technology, China

## Abstract

To evaluate the associations between candidate *FTO* single nucleotide polymorphisms (SNPs) and obesity, a case-control study was conducted among Chinese school-age children, which included 500 obese cases and 500 matched controls (age, gender and location). We selected 24 candidate *FTO* tag-SNPs via bio-informatics analysis and performed genotyping using SNPScan technology. Results indicated that rs7206790 and rs11644943 were significantly associated with obesity among school-age children in both additive and recessive models (*P*<0.05) after adjusting confounders. Comparing rs7206790 CC and CG genotype of carriers, those carrying the GG genotype had an increased risk of obesity (adjusted odds ratio [OR], 3.76; 95% Confidence interval [CI], 1.24–11.43). Carriers of the AA allele of rs11644943 had a lower risk of obesity (adjusted OR, 0.16; 95% CI, 0.04–0.72) compared with those of the T allele (TT and TA). These two SNPs (rs7206790 and rs11644943) were not Linkage Disequilibrium (LD) with previous reported obesity-associated SNPs. Under the recessive model adjusted for age and gender and location, rs7206790 GG allele carriers had significantly increased BMIs (*P* = 0.012), weight (*P* = 0.012), waist circumferences (WC) (*P* = 0.045) and hip circumferences (HC) (*P* = 0.033). Conversely, rs11644943 AA allele carriers had significantly decreased BMIs (*P* = 0.006), WC (*P* = 0.037) and Waist-to-height ratios (WHtR) (*P* = 0.012). A dose-response relationship was found between the number of risk alleles in rs7206790, rs11644943 and rs9939609 and the risk of obesity. The Genetic Risk Score (GRS) of the reference group was 3; in comparison, those of 2, 4, and ≥5 had ORs for obesity of 0.24 (95%CI, 0.05–1.13), 1.49 (95%CI, 1.10–2.01), and 5.20 (95%CI, 1.75–15.44), respectively. This study confirmed the role of *FTO* variation on genetic susceptibility to obesity. We reported two new obesity-related *FTO* SNPs (rs7206790 and rs11644943) among Chinese school-age children.

## Introduction

Obesity is characterized by the accumulation of excessive fat tissue, which may lead to adverse health consequences, such as diabetes, cardiovascular disease (CVD), and cancer [1]–[3]. The prevalence of overweight and obesity in Chinese school-age population has increased from 5.2% in 1991 to 13.2% in 2006 [4]. The growth rate of obesity in this population over the past 5 years has been 160% in urban areas and 400% in rural areas [5]–[6]. School-age obesity is associated with the risk of both adult obesity and several obesity-related chronic diseases [7]–[10]. Therefore, its prevention is important in public health.

Obesity has a multi-factorial etiology involving interactions between genetic susceptibility and environmental exposures [11]–[12]. The fat mass and obesity associated (*FTO*) gene was the first identified gene through genome-wide association studies (GWAS). *FTO* contains nine exons, and spans more than 400 Kbp. Previous studies have found that *FTO* polymorphisms rs9939609, rs1421085, rs8057044 and rs8050136 were associated with the risk of obesity, type 2 diabetes and cardiovascular disease [7]–[8], [13]–[14]. A number of studies have reported inconsistent results on the associations between genetic variants of *FTO* and the risk of obesity and obesity-related traits in both children and adults [15]–[21]. Till date, most genetic studies have focused on variants of a 42-Kbp haplotype block, around the lead single nucleotide polymorphism (SNP) rs9939609, located at the first intron. However, there have been no reports on the associations of other in *FTO* loci with the risk of obesity, particularly in school-age Asian children in China.

To examine the associations between obesity-associated genetic variations in *FTO* and the risk of obesity in Chinese school-age population, we conducted a cross- sectional case- control study and screened obesity-associated genetic variants in *FTO*.

## Materials and Methods

### Subjects

Obesity cases were defined as having a body mass index (BMI) above the 95^th^ percentile of the Chinese BMI reference data for Han children and adolescents by age and gender [22]. Normal-weight controls were defined as having a BMI between the 15^th^ and 85^th^ percentile. We recruited 500 obese cases, aged 7–18 from a cross-sectional study on metabolic syndrome that was conducted in six cities in China (Beijing, Tianjin, Chongqing, Hangzhou, Shanghai and Nanning) in 2010. Control subjects were individually matched with cases by age, gender, and location. Obese and normal-weight control subjects were unrelated. Children with cancer or other chronic diseases of the lung, heart, liver or kidney, were excluded. The study protocol was approved by the research ethics committees of the Institutional Review Board of School of Public Health, Zhejiang University and all collaborators. All participants or their legal guardians have given written informed consent.

### Physical measurements and epidemiologic investigation

Physical measurements, including waist circumference (WC), hip circumference (HC), systolic blood pressures (SBP), and diastolic blood pressures (DBP), were measured and recorded by trained investigators, following to a standard protocol. Height, weight, WC and HC (to the nearest 0.1 cm) were measured when subjects were wearing light indoor clothing without shoes. WC was measured at a level midway between the iliac crest and the lower costal margin in the standing position and at the end of a normal exhalation. HC was measured as the maximum circumference around the buttocks in the standing position. BMI was calculated as the body weight in kilograms by the square of the height in meters (Kg/m^2^). The waist-to-height ratio (WHtR) was calculated as the WC in centimeters by the height in centimeters. SBP and DBP were measured using a mercury sphygmomanometer after participants sat quietly for 15 min. The recorded of SBP and DBP values were the average of three repeat measurements after 30s intervals.

After a 12-h overnight fast, 5 ml of peripheral venous blood sample was drawn from each participant. Serum levels of total cholesterol (TC), triglyceride (TG), high density lipoprotein cholesterol (HDL-C), and low density lipoprotein cholesterol (LDL-C) were measured by enzymatic methods using a biochemical auto-analyzer (Hitachi 7060, Tokyo, Japan). Glucose was analyzed by a glucose oxidase method using the Beckman Glucose Analyzer (Beckman Instruments, Irvine, California) within 2 h of sample collection.

A face-to-face interview was conducted by trained investigators using a standardized questionnaire. Subjects were asked to provide their demographic characteristics, health status, dietary behaviors, and physical activity level, and their medical and family histories of obesity.

### Candidate SNP selection and Genotyping

Genomic DNA was extracted from the peripheral blood samples using the TOYOBO MagExtractor Genomic DNA Purification Kit (Toyobo, Osaka, Japan) following the manufacturer's protocol. A comprehensive SNP selection strategy was applied to select candidate SNPs in *FTO* [23]. We selected candidates by tagging SNPs using a targeted approach ranging from 3 kb upstream to 3 kb downstream of *FTO*, including the extensively studied rs9939609 [17]. SNPs were selected using a targeted tagging approach using the East Asians (CHB, CHD, JPT plus Asia) HapMap phase II database based on a pair-wise r^2^ of ≥0.8 among all common SNPs with a minor allele frequency(MAF) of ≥0.05. The FastSNP and SNP Function Predication softwares were used to predict the potential biological functions. Functional SNP changes includes nonsense mutations, mis-sense mutations, synonymous mutations, splicing regulation, transcription factor binding sites, enhancers, and microRNA sites; a total of 24 SNPs were selected in this study (Table 1), of which 23 were predicated to affect intron enhancement and one to affect the promoter/regulatory region. None of the candidate SNPs was in not in LD with known obesity-associated SNP (rs9939609, rs1421085, rs8057044 and rs8050136). Genotyping of the candidate SNPs was performed using SNPScan at Gene-sky Biotechnology Co. Ltd. Shanghai. In total, 499 cases and 489 controls were successfully genotyped. Repeated control samples were included in every genotyping plate with a concordance of more than 99%.

**Table 1:** Basic information of the 24 candidate *FTO* single nucleotide polymorphisms (SNPs).

SNP	Allele	Position	Functional effects	Low risk	High risk	Loci	MAF
rs13335453	T>G	52593744	Intronic enhancer	1	2	intronic	0.159
rs12596638	G>A	52673330	Intronic enhancer	1	2	intronic	0.175
rs9302654	C>T	52567046	Intronic enhancer	1	2	intronic	0.194
rs11644943	T>A	52553085	Intronic enhancer	1	2	intronic	0.226
rs16952730	A>G	52576422	Intronic enhancer	1	2	intronic	0.258
rs17236863	G>T	52699591	Intronic enhancer	1	2	intronic	0.259
rs4784323	G>A	52355066	Intronic enhancer	1	2	intronic	0.292
rs9939811	C>T	52408369	Intronic enhancer	1	2	intronic	0.297
rs6499661	C>T	52584182	Intronic enhancer	1	2	intronic	0.308
rs7206456	G>A	52562990	Intronic enhancer	1	2	intronic	0.326
rs7184897	T>A	52600965	Intronic enhancer	1	2	intronic	0.331
rs1477196	G>A	52365759	Intronic enhancer	1	2	intronic	0.341
rs741300	A>G	52691151	Intronic enhancer	1	2	intronic	0.363
rs12919344	C>A	52538175	Intronic enhancer	1	2	intronic	0.375
rs9932411	T>C	52562664	Intronic enhancer	1	2	intronic	0.376
rs1971037	C>T	52598754	Intronic enhancer	1	2	intronic	0.390
rs3751813	G>T	52376209	Intronic enhancer	1	2	intronic	0.416
rs9924072	A>G	52523564	Promoter/regulatory region	1	3	5upstream	0.420
rs3928987	G>A	52675012	Intronic enhancer	1	2	intronic	0.425
rs9939609	T>A	52378028	Intronic with no known function	0	0	intronic	0.460
rs12446047	T>C	52554803	Intronic enhancer	1	2	intronic	0.468
rs7206790	C>G	52355409	Intronic enhancer	1	2	intronic	0.491
rs708255	G>A	52680014	Intronic enhancer	1	2	intronic	0.492
rs7199716	C>T	52590749	Intronic enhancer	1	2	intronic	0.495

### Statistical analysis

Quantitative variables with normal distributions were expressed as means ± standard deviations (SD), and those with non-normal distributed variables were expressed as medians (inter-quartile range). Categorical variables were expressed as frequencies (percentages). Hardy–Weinberg equilibrium testing for each SNP in the control group was performed with the Pearson's chi-square test using the PLINK software (version 1.0.7) [24]. Statistical significance between case and controls was examined by two independent variable *t*-test for normally distributed variables or by Kruskal-Wallis tests for non-normally distributed ones. The Pearson's chi-square test was used to compare differences between categorical variables. Multivariate logistic or linear regression was used to analyze the associations between *FTO* genetic variants and obesity or obesity-related traits adjusted for potential confounders such as age, gender and location. HaploView and R for Windows 2.14.0 were used for Haplotype analysis of the 24 SNPs. The false discovery rate (FDR) approach was used to correct for multiple comparisons, using the PLINK software (version 1.0.7). The genetic risk score (GRS) was calculated as the sum of the number of risk alleles and the dose-response association was tested by the chi-square test for trend. All statistical analyses were performed using the SPSS statistical software for Windows, version 16.0 (SPSS Institute Inc., Chicago, Illinois). A *P*-value of <0.05 was considered statistically significant.

## Results

### Subject characteristics

The demographic characteristics of the 499 obese cases and 489 controls are presented in **Table 2**. The mean age was 11.8±2.8 years for cases and was 11.8±2.9 for controls (*P* = 0.85). There were no statistically significant differences between the case and control groups with regard to gender (*P* = 0.89). Cases had significantly higher BMI, WC, WHtR, SBP and DBP, TG, TC, and VLDL-C values, and lower HDL values, compared with the control group (*P* values<0.001). Diet preference (meat-dominant, balanced or vegetable-dominant), and salt preference (like, dislike, or no particular preference) were significantly difference between case and control groups (all *P* values<0.001). However, there was no difference for sweet preferences (like or dislike sweets; *P*>0.05).

**Table 2:** Characteristics of the obese and control participants.

Variables	Obese group		Control group		Total		*P*-value*
	n	Mean/median	n	Mean/median	n	Mean/median	
Gender (n, %)							0.89^b^
Male	329 (65.9%)		320 (65.4%)		649(65.7%)		
Female	170 (34.1%)		169 (34.6%)		339(34.3%)		
Age (years)	499	11.8±2.8	489	11.8±2.9	988	11.8±2.9	0.85^a^
BMI(kg/m^2^)	499	27.5±4.1	487	17.6±2.3	986	22.6±6.0	**<0.001** a
WC (cm)	497	83.3±13.3	489	60.6±8.1	986	72.0±15.8	**<0.001** a
HC(cm)	496	93.2±13.2	488	75.3±10.3	984	84.3±14.8	**<0.001** a
WHtR	497	0.54±0.06	486	0.41±0.03	983	0.48±0.08	**<0.001** a
SBP(mmHg)	495	111.1±14.5	486	101.3±11.3	984	106.3±13.9	**<0.001** a
DBP(mmHg)	497	68.8±10.2	487	63.6±7.7	984	66.0±9.4	**<0.001** a
Fasting glucose (mmol/l)	495	4.7(4.3–5.0)	487	4.7(4.4–5.0)	982	4.7(4.4–5.0)	0.76^c^
TG (mmol/l)	498	1.2(0.8–1.6)	488	0.7(0.6–1.0)	986	0.9(0.7–1.3)	**<0.001^c^**
TC (mmol/l)	497	4.2(3.7–4.7)	488	3.9(3.4–4.3)	985	4.0(3.6–4.5)	**<0.001^c^**
VLDL-C(mmol/l)	497	2.3(1.9–2.7)	488	1.9(1.6–2.2)	985	2.1(1.7–2.4)	**<0.001^c^**
HDL-C(mmol/l)	498	1.2(1.0–1.4)	488	1.4(1.2–1.7)	986	1.3(1.1–1.5)	**<0.001^c^**
Diet preference (n, %)							**<0.001^ b^**
Meat-dominant diet	111(22.3%)		52(10.7%)		163(16.5%)		
Balanced diet	339(67.9%)		388(79.3%)		727(73.6%)		
Vegetable-dominant diet	31(6.2%)		43(8.8%)		74(7.5%)		
Salty preference (n, %)							**<0.001^ b^**
like salty food	123(24.7%)		54(11.0%)		177(17.9%)		
No strong preference	309(61.9%)		371(75.9%)		680(68.8%)		
Dislike salty food	43(8.6%)		58(11.9%)		101(10.2%)		
Sweet taste (n, %)							0.40^ b^
like sweet food	294(58.9%)		322(65.8%)		616(62.3%)		
Dislike sweet food	157(31.5%)		153(31.3%)		310(31.4%)		

*P-values for differences in distribution of characteristics between the obese and control groups.

Gender was expressed as number (%); Fasting glucose, TG, TC, VLDL-C, HDL-C are expressed as median (lower quartile-upper quartile); other variables are expressed as mean ±standard deviation.

a : independent t-test; ^b^: χ^2^ test; ^c^: Kruskal-Wallis test;

Abbreviations: BMI, body mass index; WC, waist circumference; HC, hip circumference; WtHR, waist circumference to height ratio; SBP, systolic blood pressure; DBP, diastolic blood pressure; TG, triglyceride; TC, total cholesterol; LDL cholesterol, low density lipoprotein cholesterol; HDL cholesterol, high density lipoprotein cholesterol.

### FTO genetic variants and obesity

All SNPs were in Hardy–Weinberg equilibrium in both case and control groups (*P*>0.05). **Table 3** shows the associations between candidate SNPs and the risk of childhood obesity. After adjusting for age, gender and location, rs7206790 and rs11644943 significantly associated with risk of obesity in the additive and recessive models (all *P* values<0.05). When comparing the SNP rs7206790 C allele carriers (CC and CG), those with the GG genotype had increased risks of obesity with an adjusted odds ratio (OR) of 3.76 (95% confidence interval [CI]: 1.24–11.43; **Table 4**). Conversely, those that were however, homozygous for the variant allele (AA) of rs11644943 had a lower risk of obesity with an adjusted OR of 0.16 (95%CI: 0.04–0.72), compared with the T allele carriers (TT and TA). The significances did not remain after false discovery rate correction (all *P* values>0.05). Neither of the SNPs had high Linkage Disequilibrium with previously reported obesity-related SNPs such as rs9939609, rs1421085, rs8057044 and rs8050136 (all the r^2^ <0.8). False discovery rate (FDR) was used to correct for multiple comparisons test for significance, however, these significances were not remained after FDR correction (all the *P* values>0.05).

**Table 3:** Associations of the candidate *FTO* SNPs with risk of obesity in a population of school-age children.

SNP	A1	Allele		Additive model	Dominant model	Recessive model
		Obesity (499)	Control (489)	*P* a	*P* a	*P* a
rs4784323	A	26/182/291	33/179/277	0.577	0.595	0.308
**rs7206790**	G	15/139/345	4/121/364	**0.018**	0.064	**0.012**
rs1477196	A	26/181/292	23/174/292	0.896	0.702	0.714
rs3751813	T	54/216/229	40/200/248	0.189	0.121	0.160
rs9939811	T	115/247/137	117/254/118	0.489	0.233	0.744
rs9924072	G	26/178/295	27/178/284	0.939	0.740	0.828
rs12919344	A	31/187/280	39/197/252	0.280	0.149	0.280
**rs11644943**	A	3/133/363	12/124/353	**0.023**	0.805	**0.017**
rs12446047	C	25/196/278	30/195/264	0.699	0.586	0.441
rs9932411	C	27/189/283	22/187/280	0.804	0.862	0.509
rs7206456	A	48/230/221	58/215/216	0.495	0.971	0.255
rs9302654	T	6/106/387	5/104/380	0.964	0.954	0.788
rs16952730	G	39/202/258	37/204/248	0.924	0.756	0.883
rs6499661	T	2/64/433	1/45/443	0.161	0.058	0.575
rs7199716	T	77/216/206	89/221/179	0.257	0.132	0.244
rs13335453	G	30/170/299	19/165/305	0.286	0.429	0.124
rs1971037	T	113/240/146	127/233/128	0.372	0.288	0.216
rs7184897	A	107/234/158	118/235/135	0.329	0.169	0.305
rs12596638	A	63/221/215	60/219/210	0.980	0.964	0.866
rs3928987	A	76/238/185	71/242/176	0.849	0.724	0.754
rs708255	A	82/241/176	81/237/171	0.995	0.921	0.956
rs741300	G	103/250/146	98/259/132	0.642	0.429	0.815
rs17236863	T	3/38/458	2/45/442	0.615	0.442	0.670

A1: mutant allele; A2: wild-type allele.

Additive model: A1A1/A1A2/A2A2;

Dominant model: A1A1 + A1A2/A2A2;

Recessive model: A1A1/A1A2 + A2A2;

a : Logistic regression, adjusted for age, gender and location.

**Table 4:** **The** Odds ratios of rs72066790 and rs11644943 for obesity in school-age Chinese children.

SNP	Obese group(n = 499)	Control group (n = 489)	Additive model		Dominant model		Recessive model	
			OR(95%CI)	*P*-value^a^	OR(95%CI)	*P*-value^a^	OR(95%CI)	*P*-value^a^
rs7206790								
CC	345(69.1%)	364(74.4%)	1	**0.018**	1	0.06	1	**0.012**
CG	139(27.9%)	121(24.7%)	1.21(0.91–1.61)		1.30(0.98–1.71)			
GG	15(3.0%)	4(0.8%)	**3.97(1.30**–**12.10)**				**3.76(1.24**–**11.43)**	
*P* for trend	**0.02**							
rs11644943								
TT	363(72.7%)	353(72.2%)	1	**0.023**	1	0.80	1	**0.017**
TA	133(26.7%)	124(24.5%)	1.04(0.78–1.39)		0.96(0.73–1.28)			
AA	3(0.6%)	12(2.4%)	**0.16(0.04**–**0.73)**				**0.16(0.04**–**0.72)**	
*P* for trend	0.37							

A1: mutant allele; A2: wild-type allele.

Additive model: A1 A1/A1 A2/A2 A2; Dominant model: A1 A1 + A1 A2/A2 A2; Recessive model: A1 A1/A1 A2 + A2 A2;

a Logistic regression, adjusted for age, gender and location.

Abbreviations: OR, odds ratio; CI, confidence interval.

### Association between rs7206790 and rs11644943 of FTO and body measurements

The associations of body measurements with rs7206790 and rs11644943 of *FTO* are summarized in **Table 5**. Those with the rs7206790 GG genotype had significantly higher weight, WC, HC, and BMI values than the C allele carriers (all *P* values<0.05), whereas those with the rs11644943 AA genotype had significantly lower WC, WHtR and BMI values than the T allele carries (all *P* values<0.05). However, no associations were observed between the SNP genotypes and risks of other metabolic components

**Table 5:** Association of *FTO* rs7206790 and rs11644943 with body measurements.

Quantitative traits	rs7206790					rs11644943				
	CC+CG		GG		*P-*value	TT+TA		AA		*P-*value
	n	Mean ± SD	n	Mean ± SD		n	Mean ± SD	n	Mean ± SD	
height(cm)	966	150.8±16.5	19	158.0±13.2	0.073	970	151.0±16.5	15	151.4±14.6	0.861
weight(kg)	967	53.2±21.6	19	66.5±20.7	**0.012**	971	53.6±21.7	15	45.3±14.6	0.164
WC(cm)	967	71.9±15.8	19	79.2±15.1	**0.045**	971	72.1±15.9	15	65.1±11.1	**0.037**
HC(cm)	965	84.2±14.9	19	91.6±14.1	**0.033**	969	84.4±14.9	15	79.3±10.9	0.267
WHtR	964	0.48±0.08	19	0.50±0.08	0.183	968	0.48±0.08	15	0.43±0.06	**0.012**
BMI(kg/m^2^)	967	22.5±5.9	19	26.0±5.5	**0.012**	971	22.7±6.0	15	19.3±3.8	**0.006**

The *P*-value was calculated with linear regression using the additive model, adjusted for age, gender and location.

Abbreviations: WC, waist circumference; HC, hip circumference; WHtR, waist circumference to height ratio; BMI, body mass index.

No significant associations were found or rs7206790 and rs11644943 with dietary behavior (*P*>0.05).

### Genetic Risk Score and the risk of obesity

**Figure 1:**
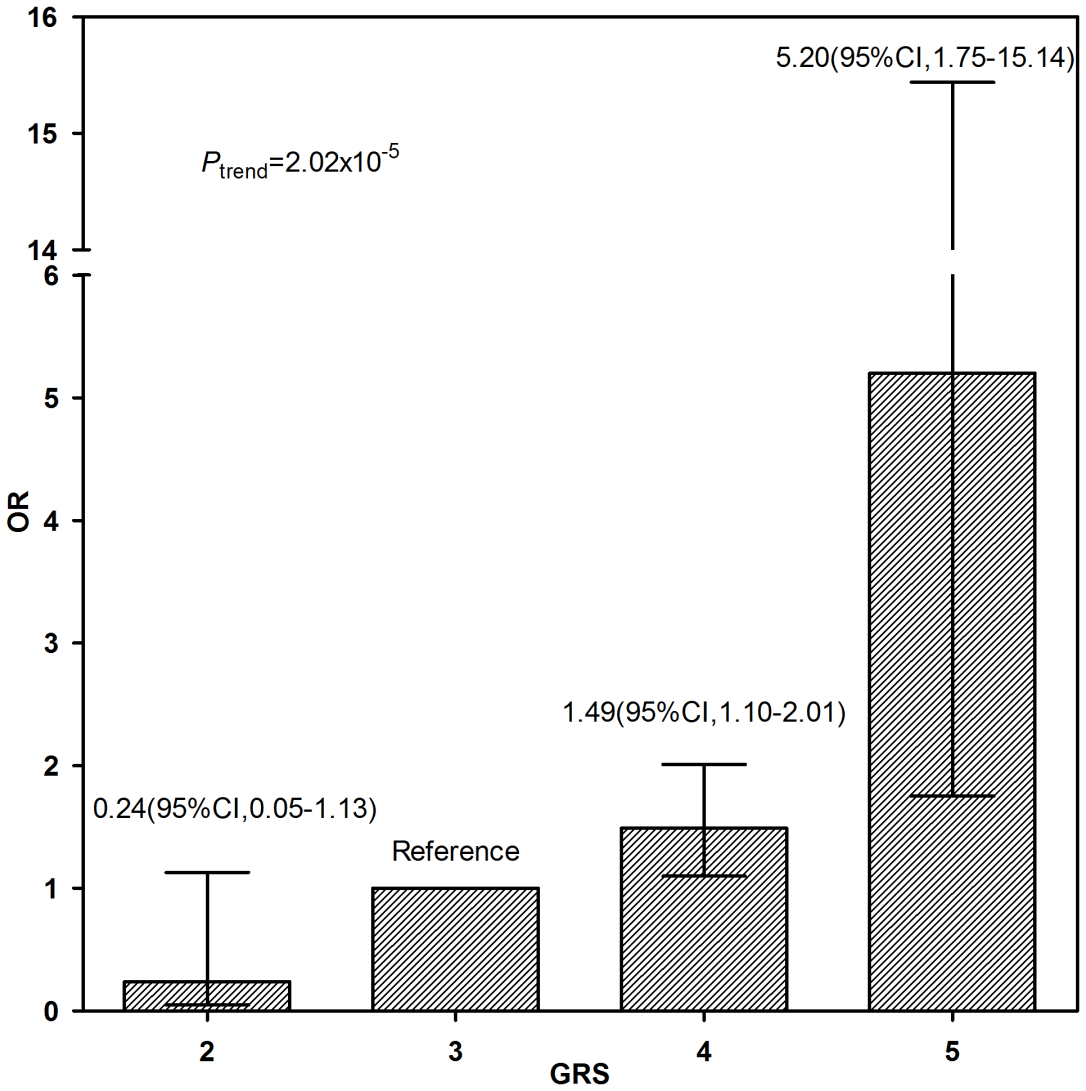
Dose-response relationship between the number of risk alleles in rs9939609, rs72066790, rs11644943 and the risk of obesity. The Genetic Risk Score (GRS) was calculated as the number of risk alleles in rs7206790, rs11644943 and rs9939609. Odds ratios and 95% confidence intervals were calculated by a logistic regression model, using the participants with GRS of 3 as the reference group.

GRS distributions, calculated as the sum of the number of the risk alleles in rs9939609, rs7206790, and rs11644943 are shown in **Figure 1**. A dose- response relationship was observed between the number of risk alleles and the risk of obesity, using a GRS of 3 as the reference group, those with a GRS  =  of 2 had a reduced the risk of obesity (OR 0.24, 95% CI: 0.05–1.13), whereas those with a GRS  =  of 4 and ≥ 5had higher risks of obesity (OR 1.49, [95% CI: 1.10–2.0] and 5.20 [95% CI: 1.75–15.44], respectively.

## Discussion

In this study, we systematically assessed the obesity-related genetic variations in *FTO* in a population-based case-control study. Consistent with previous studies, we replicated the association of SNP rs9939609 with both BMI and obesity in Chinese school-age children. In addition, two novel SNPs (rs7206790 and rs11644943) were found significant associated with the risk of childhood obesity. Together with rs9939609, dose-response relationships existed between the number of risk alleles and the risk of obesity. These findings indicate that genetic variation of *FTO* was associated with the risk of obesity, and that rs7206790 and rs11644943are novel obesity-related genetic variants among Chinese school-age children.

Obesity is resulted from interaction of genetic susceptibility and environmental exposures. Genetic susceptibility plays a vital role in increasing the risk of obesity. *FTO* gene was the first identified gene through GWAS. Several studies have evaluated the associations between some SNPs (rs9939609, rs1421085, rs8057044 and rs8050136) in *FTO* gene and risk of obesity and obesity-related traits in both children and adults, but the results remain inconsistent [25]–[28]. Although *FTO* has emerged as a major obesity-related gene particularly in populations of European descent [16]–[17], results in Asian populations are inconclusive. Indeed, no significant association was found between *FTO* polymorphisms and obesity in Chinese Han population [29], which was inconsistent with subsequent studies in Taiwan [30]and Beijing [31]. In our case-control study, we recruited 1000 Chinese school-age children from northern, central, and southern regions of China. The associations of *FTO* polymorphisms rs9939609 with the risk of obesity and obesity-related traits were confirmed and the influence of rs9939609 on dietary behaviors (e.g., preference for a meat-based diet and salt and sweet tastes) was evaluated to in an attempt account for any observed physical and metabolic association (Results were reported in another article which was completed by our research team). In this case-control study, two novel SNPs (rs7206790 and 11644943) were significantly associated with the risk of obesity and obesity-related traits among Chinese school-age children. The inconsistent results may be because of the heterogeneity of the study populations, different definitions of obesity, or the confounding effects of environmental variables. Participants in the present study were school-age children from 7 to 18 years, whereas adult women aged from 50 to 70 years were included in another study [32]. In a European population, Loos et al. found that the association between *FTO* and obesity was more significant in children than in adults [33]. This suggests that younger participants are less affected by environmental factors on obesity, thus highlighting the role of genetic factors. With increasing age, the cumulative effects of environmental factors may gradually override the role of genetic factors on the development of obesity.

Additionally, we found that rs7206790 was significantly associated with the risks of obesity and several obesity-related metabolic traits (BMI and HC), which was similar to the study by Seongwon Cha [34]. Although the results were not statistically significant in other studies [35]–[36].We confirmed that *FTO* rs9939609 was strongly associated with obesity-related metabolic traits such as WC, fasting glucose, LDL-C and HDL-C after adjusting for age, gender and location. We also found that SNPs rs7206790 and rs11644943 were associated with several obesity-related metabolic traits such as BMI and HC. These findings suggest that the risk alleles of *FTO* SNPs contribute to the higher BMI and HC and to the occurrence of obesity in the school-age Chinese Han population. Previous studies have observed similar results [29], [37], but the mechanisms by which *FTO* SNPs influence obesity and obesity-related metabolic traits remains unclear. Further functional studies are required.

Obesity, a human common complex disease, is resulted from interaction of gene-environment. Studies have found individuals have diverse food selection and energy intakes because of the combined effects of genetic features (resulting in metabolism and physiological differences) and lifestyle behaviors. The associations between *FTO* SNPs and obesity may be explained by their effects on dietary behaviors. Increasing energy intake is a major determinant of the current obesity epidemic. A preference for high-energy foods induced by *FTO* SNP variations, may partially explain the predisposition to obesity. Zhifu Han, et al. reported the crystal structure of the *FTO* protein reveals basis for its substrate specificity, which was in complex with the mononucleotide 3-meT [38]. The data provided structural evidence to support the notion that *FTO* can act as a DNA/RNA demethylase for its functions. *FTO* may affect fat metabolism by influence the stability of the modified ribosome, since the main characteristics of mononucleotide 3-meT was to combine with single nucleic acid in the body. Nevertheless, this structural information provides a starting point for the successful development of *FTO* inhibitors that holds promise for developing therapeutic agents to treat obesity. Future studies are needed to determine how they contribute to substrate recognition by *FTO*. Previous study also showed that *FTO* was widely expressed in fetal and adult tissues, with the highest expression found in the brain tissue, which is a key controller of energy balance; thus, variation in expression may result in carriers of the risk allele (A) and develop obesity through excessive ingestion rather than altered energy consumption. Similar results have been reported in previous studies [39]–[40]. Cecil and colleagues reported that the A allele of *FTO* rs9939609, which has been linked with obesity, was also associated with the control of food intake and food choice in children. Children carrying the A allele intake more energy-dense foods than those carried homozygote wild type (TT), suggesting a link to a hyperphagic phenotype or a preference for energy-dense foods.

No significant associations were found for rs7206790 and rs11644943 with diet preference (meat-dominant, balanced or vegetable-dominant), salt preference, and sweet preference (all *P*>0.05), and no combined effects were observed for other *FTO* SNPs and dietary behaviors on obesity. However our results suggest that AA homozygous rs9939609 cases were more likely to choose a meat-based diet, which has a higher energy intake than either vegetable-based or balanced diets, compared with the other two genotypes. As discussed earlier, high energy intake may explain the predisposition to obesity and may occur through a meat-based diet. Although no significant associations were observed for dietary behaviors in our study, previous studies have demonstrated the significant effects on obesity [41]–[42]. This inconsistency may be explained by the heterogeneity of study subjects and differences in the quantitative criteria of sweet foods. These results indicate that dietary behaviors play an important role in the development of obesity, and that at-risk children can reduce their risk of later obesity through healthy dietary behaviors such as eating more vegetables and having a preference for mild taste [43]. Obesity is a complex disease affected by multiple genes and environmental factors, and further investigation is requires to verify most relevant interactions in children.

This case-control study has several strengths. First, our study was a multi-center collaborative study, with participants recruited from a representative population of Chinese school-age children. Second, we used standardized methods to identify childhood obesity. Third, all SNPs selected in our study had biological implications and some had previously been associated with obesity [16]–[17]. Fourth, we systematically evaluated the impact of *FTO* polymorphisms on dietary behaviors (preference for a meat-based diet and salt and sweet tastes), and our findings may explain how *FTO* variations confer a predisposition to obesity. Finally, we adjusted for a number of confounding factors in the multivariate model including age, gender and location. Despite the strengths, our study had several limitations. A major limitation was that we only included school-age Chinese children, so we cannot extrapolate our findings to other ethnic groups. Therefore, further evaluation is needed in other populations to confirm our findings. Additionally, information about dietary behaviors was self-reported in our study. The data would be more reliable if food intake were more accurately recorded with diet diaries. However, such recording is difficult in epidemiological surveys with relatively large samples. Although we controlled for key confounding factors such as age, gender, and location, but other potential confounders were not considered because of the high proportion of missing data; such confounders include socioeconomic status, pubertal status, and physical activities and energy intake. Finally, the number of individuals with relevant mutations who preferred a healthy-risk diet (i.e., meat-based diet and salt and sweet tastes) was very small because of the low frequency of variant alleles in the population. Hence further studies are needed in a larger population.

In conclusion, in this study, we confirmed the role of *FTO* on genetic susceptibility to obesity and discovered two new *FTO* obesity-related SNPs (rs7206790 and rs11644943), in Chinese school-age population. The roles of these two SNPs should be validated in larger populations and with function assays.
